# In Vitro Evaluation of Nematodicidal Bacteria Isolated from Cow Dung Against Strongyle Nematodes of Small Ruminants

**DOI:** 10.3390/pathogens15101035

**Published:** 2026-10-01

**Authors:** Dhandapani Kuppusamy, Ponnudurai Gurusamy, Rani Natarajan, Selvaraju Ganapathy, Ponnusamy Periyasamy, Srinivasan Palani, Saravanan Ramasamy

**Affiliations:** 1 Department of Veterinary Parasitology, Veterinary College and Research Institute, Namakkal 637 002, Tamil Nadu, India; king.kdhan@gmail.com; 2 Veterinary College and Research Institute, Theni 625 534, Tamil Nadu, India; 3 Department of Veterinary Parasitology, Veterinary College and Research Institute, Udumalpet 642 205, Tamil Nadu, India; ranideepan99@gmail.com; 4 Department of Veterinary Public and Health, Veterinary College and Research Institute, Namakkal 637 002, Tamil Nadu, India; selvabhuvana@gmail.com; 5 Department of Veterinary Microbiology, Veterinary College and Research Institute, Namakkal 637 002, Tamil Nadu, India; ponsvhl@gmail.com; 6 Poultry Disease Diagnosis and Surveillance Laboratory, Veterinary College and Research Institute Campus, Namakkal 637 002, Tamil Nadu, India; srinivasan.p@tanuvas.ac.in; 7 Department of Animal Husbandry Statistics and Computer Applications, Veterinary College and Research Institute, Namakkal 637 002, Tamil Nadu, India

**Keywords:** nematodicidal bacteria, biological control, gastrointestinal nematodes, larval feeding assay, simulation pasture model study, strongyle nematodes, small ruminants

## Abstract

A study was carried out to identify the nematodicidal bacteria in ruminant dung and assess their efficacy against gastrointestinal nematodes of small ruminants. A total of 10 bacteria were isolated from ruminant dung and their nematodicidal activity was determined. Evaluation of nematodicidal efficacy of all the isolates through larval feeding assay revealed 0.00 ± 0.00 to 100.00 ± 0.00% efficacy with varying time. However, of the 10 bacterial isolates from the dung, three bacterial isolates showed 100.00 ± 0.00% nematodicidal activity in 4 h of incubation. The bacterial isolates were preliminarily classified based on cultural and Gram-staining characteristics and were subsequently identified by 16S rRNA gene sequencing. Molecular analysis confirmed that the three isolates exhibiting the highest nematodicidal activity in the larval feeding assay were *Citrobacter amalonaticus*, *Shigella flexneri*, and *Klebsiella aerogenes*. The isolate *Citrobacter amalonaticus* (5.5 × 10^8^ CFU per mL) caused 100% larval mortality at 10 and 7.5% culture broth concentrations in 4 and 6 h of incubation, respectively in a dose-dependent larval assay. In the simulation pasture model study, compared to the control tray, the pasture tray treated with the bacterium *Citrobacter amalonaticus* (5.6 × 10^8^ CFU per mL) showed a reduction in larval recovery of 87.50%. The present work using bacteria isolated from animal origin against nematodes affecting small ruminants is the first of its kind in India. The results of this seminal work will go a long way in developing an alternative strategy for chemical control of the worm problem in animals.

## 1. Introduction

India possesses the largest livestock population in the world (535.78 million animals). Of the approximately two billion in the world’s livestock population, small ruminants alone account for 50 per cent. According to the 20th livestock census conducted in India), the small ruminant population has increased by 24% compared to the previous census. The small ruminant population in Tamil Nadu accounts for more than six per cent of the country’s total population of small ruminants and contributes significantly to the national economy. Sheep and goat rearing is one of the important farming practices along with agriculture in arid and semi-arid regions. Sheep and goat farming are becoming popular among small and marginal farmers because it can adapt to varying climatic conditions.

In recent years, gastrointestinal nematode (GIN) infection has become a major constraint to the efficient production of small ruminants, causing substantial economic losses at multiple levels. The direct economic losses include clinical morbidity, mortality and increased cost of health management and indirectly cause reduction in weight gain, decreased milk production and poor reproductive performance. The economic losses due to GIN infection in small ruminants in India are estimated to be 103 million USD per annum [1]. In Tamil Nadu, the economic loss due to GINs in small ruminants has been estimated at rupees 1000 to 1600 loss/animal [2].

Control measures against GINs in sheep and goats are usually achieved with chemical anthelmintic drugs along with nutritional and pasture management. Although these drugs have been effectively used in the past to control GIN infection and increase the productivity of grazing ruminants, the emergence of drug resistance to anthelmintics has become a significant concern worldwide [3]. Anthelmintic resistance has been reported in several nematodes affecting small ruminants, including *Haemonchus* spp., *Trichostrongylus* spp., *Oesophagostomum* spp., *Teladorsagia* spp., and *Cooperia* spp. [4]. Besides resistance, concerns regarding drug residues in meat and milk, environmental pollution, and treatment costs have limited the sustainability of chemical control strategies. As a result, greater emphasis is being given to alternative and supplementary control methods.

In recent years, biological control using bacteria and nematophagous fungi has emerged as a promising alternative for managing plant and animal parasitic nematodes. Several species of bacteria isolated from soil, vegetation and animal dung have demonstrated nematicidal activity. Their efficacy is attributed to successful niche colonization, competition and the production of nematicidal and antimicrobial compounds [5]. Plant-parasitic nematodes (PPNs), such as cyst nematodes (*Heterodera* and *Globodera* spp.), root-knot nematodes (*Meloidogyne* spp.), lesion nematodes (*Pratylenchus* spp.), reniform nematodes, and dagger nematodes (*Xiphinema* spp.), are widely distributed and cause substantial damage to plants [6]. In addition, stem- and leaf-feeding nematodes, including *Aphelenchoides* and *Bursaphelenchus* spp., are important plant parasites. Several workers have investigated different approaches for the effective management of PPNs, with particular emphasis on bacterial biocontrol agents including *Bacillus atrophaeus*, *Pseudomonas simiae*, *Serratia ureilytica*, *Pseudomonas fluorescens*, *Serratia plymuthica*, *Bacillus firmus*, *Bacillus thuringiensis*, *Pasteuria* sp. Ph3, *Pseudomonas rhodesiae*, *Streptomyces microflavus* A12 [7,8,9,10,11,12,13,14,15,16,17,18]. Among these, *Pasteuria pentrans*, *Bacillus* spp., *Pseudomonas*, and *Serratia* spp. have demonstrated considerable nematicidal efficacy over the past two decades, leading to the development of commercial bacterial nematicidal preparations for field-level application [11,17,18].

Despite the growing interest in biological control, studies focusing on bacteria as biological control agents against animal nematodes are found to be scanty, highlighting a significant gap in biocontrol research. Only a limited number of bacterial species, such as *Chryseobacterium nematophagum*, *Comamonas testosteroni*, *Comamonas jiangduensis*, and *Pseudomonas weihenstephanensis*, have demonstrated nematodicidal activity [19,20]. Hence, the present study was undertaken to isolate nematodicidal bacteria from dung and evaluate their efficacy against GINs of sheep. This approach primarily aims to reduce the number of infective L_3_ available to the grazing animals; thereby, GIN infection-associated economic losses can be minimized to a larger extent.

## 2. Materials and Methods

The research work was conducted at the Department of Veterinary Parasitology, Veterinary College and Research Institute, Namakkal, India. The area is located at 11.16° N, 78.16° E and an altitude of 218 meters above sea level.

### 2.1. Sample Collection

The dung samples were collected per rectum from the cattle maintained under a semi-intensive rearing system in the northwestern zone of Tamil Nadu and transferred aseptically into sterile containers. The sampling location is situated at approximately 11.29° N latitude and 78.01° E longitude, with an elevation of approximately 218 m above mean sea level.

### 2.2. Isolation of Bacteria from Dung

One gram of the fecal sample was transferred into a sterile stainless-steel container and mixed with 10 mL of sterile distilled water to prepare a homogeneous fecal solution. Thereafter, 1 mL of the homogenized fecal solution was transferred into a sterile centrifuge tube containing 9 mL of sterile distilled water to obtain a tenfold dilution. A 1 mL aliquot of the 10^−2^ diluted fecal solution was transferred into the nutrient broth and incubated overnight at 37 °C. After incubation, a loop of bacterial culture was streaked onto nutrient agar (Himedia, India), MacConkey agar (Himedia, India), Mannitol salt agar (Himedia, India), and brain–heart infusion agar (Himedia, India). All the plates were incubated at 37 °C overnight. Representative individual colonies were isolated as pure cultures by streaking onto specific agar media. The purified isolates were used for a larval feeding assay. All the isolated bacteria were stained with Gram stain and identified. The isolated bacteria were stored in 80% glycerol broth at −80 °C for molecular assay.

### 2.3. Mass Harvesting of Strongyle Eggs for In Vitro Assay

Approximately 500 g of pooled dung sample was collected per rectum from randomly selected adult sheep (8 to 12 months old) within the sheep flock. The dung was kneaded with gloved hands to get a uniform mixture, and then 2 L of water was added to dissolve it. The fecal suspension was filtered through mesh flour sieves. The filtered solution was subjected to a simple sedimentation process for 30 min to allow the contents to settle at the bottom. Subsequently, the clear supernatant liquid was carefully decanted. The sediment was transferred to a beaker and 10 volumes of saturated salt solution were added. The mixture was left undisturbed at room temperature (35–37 °C) for 10 min, after which the superficial supernatant was carefully transferred to a clean beaker. The supernatant was then diluted with 10 volumes of tap water and allowed to settle for 1 h. The supernatant was discarded and the sediment was transferred to polyallomer tubes. Saturated salt solution was added to 2/3rd of the tube volume, followed by centrifugation at 2000 rpm for 2 min. After centrifugation, the superficial upper layer was separated by clamping with artery forceps and transferred into a fresh centrifuge tube, diluted to 14 mL with water and centrifuged at 2000 rpm for 2 min. The aforementioned washing procedures were repeated three times to obtain a final suspension volume of 1 mL. A 10 µL aliquot of the suspension was examined under a light microscope. Finally, the solution was adjusted to contain 200 eggs per 100 µL.

### 2.4. In Vitro Larval Feeding Assay

The larval feeding assay was initially performed as a primary screening assay to evaluate the nematodicidal potential of the isolated bacterial strains. One hundred microliters of egg suspension was transferred to each well in the 24-well tissue culture plates. Subsequently, 1700 µL of distilled water was added to all the wells and incubated overnight at room temperature (28–37 °C). Thereafter, after 18 h of incubation, 200 µL (10% of the total volume) of pure bacterial culture was added to all the wells except the control wells, for which the same quantity of water was added. An equal volume of probiotic preparation containing Lactobacillus culture was added to the positive wells. The plate was examined under an inverted tissue culture microscope (10 and 45X) to determine larval viability at 0 min, 30 min, 1 h, 2 h, 4 h and 6 h after addition of the pure bacterial culture. The plate was incubated at room temperature and the final larval count was recorded 24 h after the addition of the pure bacterial culture. Larvae were considered dead when they remained immotile and showed no response to gentle shaking or mechanical stimulation with a fine needle. For each isolate, the assay was carried out in triplicate, and the average was calculated using the following formula.

Larval mortality = (Dead larva/Total larva) × 100

### 2.5. Identification of Bacteria by Molecular Assay

In this study, bacterial isolates that showed 100% nematodicidal activity were identified by 16S rRNA gene sequencing. The DNA was extracted from overnight pure cultured broth using a SCITEOGEN bacterial DNA isolation kit (India) and amplified with universal bacterial primers 27F and 1492R [21]. PCR amplification was carried out for 30 cycles with denaturation at 95 °C for 1.5 min, annealing at 55 °C for 1.5 min, and extension at 72 °C for 1.5 min. The PCR products were subjected to Sanger’s sequencing method and the results were analyzed with NCBI BLAST. Phylogenetic analysis was performed using MEGA 11 software, and the phylogenetic tree was constructed using the Maximum Likelihood method with the Tamura–Nei nucleotide substitution model and 1000 bootstrap replicates.

### 2.6. Dose-Dependent Maximum Larval Mortality Study

During the primary assay, those bacterial strains that exhibited rapid nematicidal activity were selected for further evaluation. The maximum larval mortality rate was determined for each concentration (10%, 7.5%, 5%, 2.5%, and 1.25%) of the selected bacterial culture broth. The bacterial culture broth was subjected to standard plate count analysis to determine the viable bacterial cell concentration, which was expressed as colony-forming units per mL (CFU/mL). The dose-dependent maximum nematodicidal effect was assessed using 10, 7.5, 5.0, 2.5, and 1.25% pure bacterial culture broth. Approximately 200 nematode eggs suspended in 100 µL were dispensed into each well of a 24-well tissue culture plate, followed by the addition of 1700 µL of sterile distilled water. After overnight incubation (18 h) at room temperature, 200, 150, 100, 50, and 25 µL of pure bacterial culture were added to obtain the respective concentrations, and the final volume was adjusted with sterile distilled water. The larval count was monitored using an inverted tissue culture microscope (10 and 45X) at 0 min, 30 min, 1 h, 2 h, 4 h and 6 h after the addition of the pure bacterial culture. The plate was incubated at room temperature and a final larval count was performed 24 h after the addition of pure bacterial culture. For each concentration, the assay was carried out in triplicate, and the average was calculated using the previously described formula.

### 2.7. Simulation Pasture Model Study

Nutrient-rich nursery soil was sun-dried for 7 days and sterilized by autoclaving at 120 °C for 30 min. The treated soil was evenly distributed to a depth of 3 inches in two plastic trays (500 cm^2^), designated as the control and treatment trays. Sudan grass seeds were sown and irrigated for 10 days. The grass was used for the experiment after reaching a height of 8 to 10 cm. During the experiment, the average temperature was about 28.4 °C and the humidity was 66.7%.

Nematode eggs were recovered from fecal samples following the previously described procedure (see Section 2.3). The egg suspension was diluted with sterile distilled water to obtain a final concentration of 1000 eggs/mL and was subsequently used in the experiment.

On the 10th day after sowing, 10 mL of egg suspension was sprayed on both grass plates. On the 11th day, an overnight incubated bacterial culture broth (CFU cell/mL) was sprayed onto the experimental tray (Figure 1). Ten days after bacterial application, 5 g of herbage was harvested from six randomly selected locations above the soil surface. The grass samples were immersed in 1% detergent solution for 8 h at room temperature. The sediment obtained after incubation was centrifuged at 2000 rpm for 3 min, and the recovered larvae were counted under a low-power light microscope. The final result was obtained by the following formula:

L_3_/Kg of DM= Number of L_3_ × 1000/Weight of the grass (DM)

L_3_ = Third-stage larva; DM = Dry Matter

**Figure 1:**
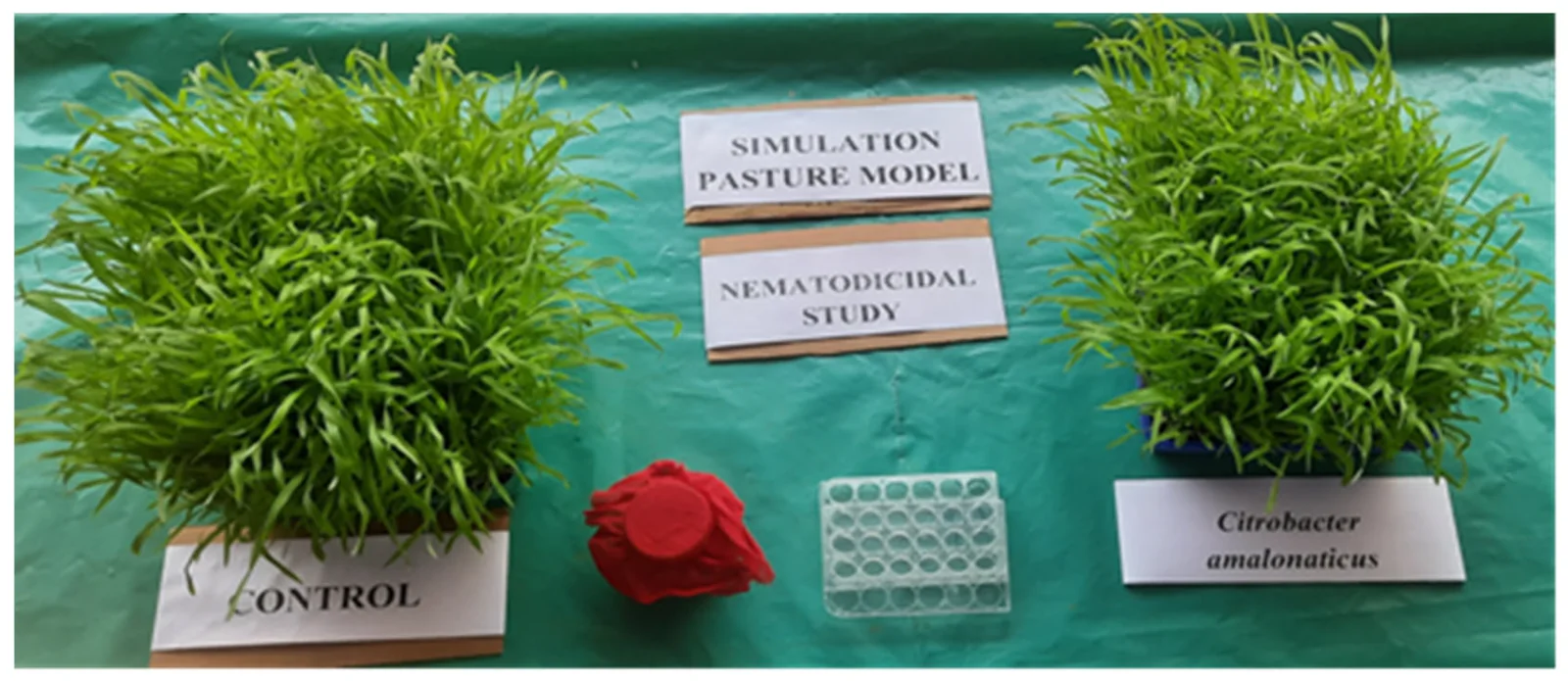
Simulation pasture model—*Citrobacter amalonaticus*.

### 2.8. Statistical Analysis

The data that were collected in this study were analyzed by Statistical Package for the Social Science (version 20 for Windows, SPSS) software. Comparative values of nematodicidal effects of different isolates were analyzed by one-way ANOVA and Post Hoc Test (Duncan test, *p* < 0.05). The LC_50_ values were calculated using probit analysis of percentage larval mortality recorded at different concentrations after 24 h of exposure, using SPSS software (version 20 for Windows, SPSS).

The relationship between bacterial culture concentration and larval mortality was evaluated using concentration-wise dose–response regression analysis at each exposure time. Bacterial culture concentration (%) was included as the predictor variable and larval reduction (% mortality) as the response variable. Regression models were fitted separately for 30 min, 1 h, 2 h, 4 h, 6 h and 24 h of exposure. Both linear and second-order polynomial regression models were evaluated, and the coefficient of determination (R^2^), adjusted R^2^, regression coefficients, standard errors and significance levels were used to assess the strength and nature of the concentration–response relationship.

The fitted model was:

Y = a + bX + e

where Y = larval reduction (%); a = constant; b = bacterial culture concentration.

## 3. Results

### 3.1. Bacterial Isolation

Well-isolated colonies with distinct morphological characteristics were selected from each agar plate and examined by Gram staining. Based on their cultural and Gram-staining characteristics, ten bacterial isolates, comprising seven Gram-Negative and three Gram-Positive bacteria, were identified from the four culture plates. A total of ten isolates were isolated from cattle dung. The morphological and cultural characterizations of bacterial isolates are presented in Table 1 and Figure 2.

### 3.2. Bacterial Isolates for Nematodicidal Activity by Larval Feeding Assay

The nematodicidal activity of the bacterial isolates was evaluated using an in vitro larval feeding assay. During the primary screening, exposure to 10% bacterial culture broth resulted in complete larval mortality (100%) in six isolates, 94–99% mortality in three isolates and 0% mortality in one isolate after 24 h of incubation. The maximum percentage of larval mortality was observed in the four isolates at 4 h, and no larval recovery was observed even at 48 h.

The results revealed that considerable variation was observed in the nematicidal activity of bacterial isolates, ranging from 0.00 ± 0.00 to 100.00 ± 0.00% at 24 h incubation. The bacterial isolates of C2MCPK, C2MCPL and C2BHSY exhibited 100 00 ± 0.00% larval mortality in 4 h of incubation. Similarly, the C1MCPK, C2NIC and C2BHIC isolates produced 100.00 ± 0.00% larval mortality in 6 h of incubation. The remaining active isolates, C1NIC, C1MCPL, C2NTR and C2MS, reached their maximum larvicidal activity after 24 h incubation with mortality rates of 97.33 ± 2.18, 94.00 ± 1.00, 99.00 ± 0.00 and 0.00 ± 0.00% respectively (Table 2).

All the isolates showed an increase in larval mortality with increasing time from 0 to 6 h. No larval recovery was observed in any of the bacteria even after 48 h of incubation. There was a significant (*p* < 0.05) difference in the percentage of nematodicidal effects between each isolate positive (Probiotic) and negative control (Water). In the negative control, larval mortality of up to 2.33 ± 0.67% was observed in 24 h of incubation. In the positive control, larval mortality of up to 0.0 ± 0.0% was observed in 24 h of incubation.

### 3.3. Molecular Analysis and Identification of Bacterial Isolates

A total of three bacterial isolates were selected (C2MCPK, C2MCPL and C2BHSY) that exhibited high nematodicidal efficacy, were amplified by PCR for species-level identification and an amplicon size of~1400bp was obtained. The amplicon of each product was sequenced and compared with the sequence available in the NCBI database. The identified isolates were confirmed by phylogenetic analyses based on 16S rRNA gene sequences and are listed in Figure 3. The phylogenetic tree analysis demonstrated that the bacterial isolates in the present study clustered with *Citrobacter amalonaticus* (84%), *Shigella flexneri* (96%) and *Klebsiella aerogenes* (89%).

### 3.4. Dose-Dependent Maximum Larval Mortality

Based on the results of the primary screening assay, one bacterial (C2MCPK) isolate was selected from the three isolates that achieved 100% nematodicidal activity within 4 h for further evaluation of a dose-dependent larval mortality study. The result of the standard plate count of the *Citrobacter amalonaticus* culture broth was found to be 5.5 × 10^8^ bacterial cells per mL. The results of the concentration-based study of *Citrobacter amalonaticus* are presented in Table 3. Larval mortality was observed to be 100.00 ± 0.00, 98.67 ± 0.88, 93.33 ± 0.33, 40.00 ± 0.58 and 14.00 ± 2.31% at 10, 7.5, 5, 2.5 and 1.25% concentrations of culture broth respectively at 4 h of incubation, respectively. However, 7.5% concentration also produced 100%larval mortality at 6 h of incubation. In contrast, a low level of 97.67 ± 2.33, 21.67 ± 2.33 and 15.67 ± 2.03% larval mortality was observed at 2.5 and 1.25 per cent, respectively.

The larval mortality recorded in the 10% and 7.5% treated groups significantly differs (*p* < 0.05) from the low concentration and the control. In the control, larval mortality of 12.33 ± 2.60% was observed at 24 h of incubation. The LC_50_ value of the culture broth against larvae was 55.12 μL (2.5%) after 24 h exposure.

### 3.5. Dose–Response Regression Analysis

Regression analysis demonstrated a significant concentration-dependent increase in larval reduction at all exposure periods. The linear regression model showed a positive association between culture concentration and larval reduction, with coefficients of determination (R^2^) ranging from 0.453 at 30 min to 0.922 at 2 h. The strongest linear relationship was observed at 2 h (R^2^ = 0.922, *p* < 0.001).

Because larval reduction approached 100% at higher bacterial culture concentrations and longer exposure periods, a second-order polynomial regression model was additionally fitted to account for the apparent non-linear dose–response relationship. The quadratic model provided a substantially better fit at 2 h, 4 h, 6 h and 24 h. The highest goodness of fit was observed at 4 h (adjusted R^2^ = 0.967), followed by 2 h (adjusted R^2^ = 0.952). The linear and second-order polynomial models were compared using Akaike Information Criterion (AIC), adjusted R^2^, and residual error. Model selection was based on the relative Akaike Information Criterion (AIC) values, with lower AIC indicating a better balance between model fit and complexity. The quadratic term was statistically significant at 2 h (*p* = 0.0029), 4 h (*p* < 0.001), 6 h (*p* = 0.011) and 24 h (*p* = 0.010), indicating a curvilinear concentration–response relationship. These findings demonstrate that increasing bacterial culture concentration markedly enhanced larval reduction, with the response progressively approaching a maximum at higher concentrations.

### 3.6. Simulation Pasture Model Study

A simulation pasture model analysis demonstrated the nematodicidal activity of *Citrobacter amalonaticus* (5.6 × 10^8^ CFU per mL). A low larval burden of 300 L_3_ per kilogram of dry matter was observed in the treated tray compared with 2400 L_3_ per kilogram of dry matter in the control tray (Figure 4).

## 4. Discussion

Sheep and goat rearing is an integral component of livestock in developing countries, providing a dependable source of income and food security among smallholder and marginal farmers. However, productivity and profitability are severely constrained by gastrointestinal nematode infection, which results in considerable economic losses, poor feed conservation, decreased reproductive performance and increased mortality. The control of gastrointestinal nematodes in small ruminants primarily relies on the use of synthetic anthelmintic drugs. However, their indiscriminate use has promoted the widespread development of anthelmintic resistance among nematode populations. In addition, concerns regarding drug residues in animal products and environmental contamination have highlighted the limitations of conventional drug control. Consequently, there is a need to develop safe, sustainable and environmentally friendly alternative strategies for the control of gastrointestinal nematodes in small ruminants.

In recent years, biological control has gained considerable attention as an alternative strategy for the management of gastrointestinal helminths. Among the biological agents bacteria and nematophagous fungi have shown promising potential. Although numerous bacterial species isolated from soil and plant sources exhibit strong nematicidal activity against plant-parasitic nematodes, studies evaluating their efficacy against gastrointestinal nematodes in livestock remain limited. This knowledge gap underscores the need to identify and evaluate new bacterial isolates for the biological control of animal-parasitic nematodes.

In the present study, the nematodicidal activity of bacteria isolated from cattle dung was evaluated against the sheep and goat strongyle group of nematodes. A total of 10 isolates were identified and their nematodicidal activity was determined. The dose-dependent nematodicidal activity of *Citrobacter amalonaticus* was also tested through a larval feeding assay. The highest larval mortality was recorded at the concentrations of 10 and 7.5% within 4 to 6 h intervals. Similarly, *Citrobacter amalonaticus* showed a significant reduction in L_3_ in simulation pasture model studies.

In this research, a total of ten bacterial isolates were isolated from the ruminant dung and tested through a larval feeding assay. The results of the study are in consonance with previous research; the findings identified 17 potential nematodicidal bacteria from cow dung in China [22]. However, all the bacterial isolates isolated by the authors were different from the isolates recorded in the present study. Similarly, earlier work isolated three bacterial isolates from the abomasal content of sheep in their study, but the isolated bacterial isolates also differed from bacterial isolates of this study [20]. This variation in the bacterial isolates could be due to geographical variations, isolation methods, and climatic conditions.

Although larval mortality began within 30 min in a few isolates, 100% larval mortality was observed after 4 h of incubation in three isolates, while another three caused 100% larval mortality after 6 h of incubation. A total of seven isolates caused 100% larval mortality in 24 h. It was generally observed that larval mortality increased with increasing exposure time. The present findings are consistent with previous reports showing that *Comamonas testosteroni*, *Comamonas jiangduensis*, and *Pseudomonas weihenstephanensis* produced 100% larval mortality against *Haemonchus contortus* L_3_ larvae within 3 h of incubation [20]. Similarly, 17 bacterial strains were previously reported to cause more than 90% larval mortality against the model nematode *Caenorhabditis elegans* and the plant-parasitic nematode *Meloidogyne incognita* within 1 h of incubation [22]. It is noteworthy to mention that variation in the time taken by the isolates to produce 100% mortality could be due to variation in the type of compounds produced by the isolates that cause larval death and the nematode species used for the study.

In the current study, the sequence analysis of the 16S rRNA gene identified the bacterial isolates with potent nematodicidal activity: *Citrobacter amalonaticus*, *Shigella flexneri* and *Klebsiella aerogenes*. Similar amplification profiles and sequence identities have also been documented and identified for different bacterial isolates obtained from soil, animal and plant sources [23,24,25,26,27]. Reports on bacterial species exhibiting nematodicidal activity against strongyle nematodes of ruminants remain scarce. Previously identified species include *Bacillus thuringiensis*, *Bacillus circulans*, *Comamonas testosteroni*, *Comamonas jiangduensis*, *Pseudomonas weihenstephanensis*, and *Chryseobacterium nematophagum* [20,28,29]. The identification of *Citrobacter amalonaticus*, *Shigella flexneri*, and *Klebsiella aerogenes* in the present study broadens the spectrum of bacterial species with demonstrated nematodicidal activity and highlights their potential as biological control agents against gastrointestinal nematodes of ruminants.

In this investigation, one isolates those that have been selected that possess the highest nematodicidal activity. *Citrobacter amalonaticus* was subjected to a dose-dependent larval feeding assay. In *Citrobacter amalonaticus*, 100% larval mortality was observed at a 10% concentration of culture broth after 4 h of incubation, while complete mortality was observed after 6 h of incubation at 7.5% concentration.

The findings of the present study are in corroboration with previous reports which demonstrate a similar dose-dependent activity and a lower rate of larval mortality as the decreasing concentration of bacterial metabolite [20]. It is important to emphasize that the authors recorded 100% larval motility only at 100% concentration of the bacterial metabolite used in their study. In contrast, in the present study 100%larval mortality was achieved at the lower concentrations of 10 and 7.5%. This observation points to marked nematodicidal effects possessed by the isolates of the present study compared to the isolates of earlier studies.

The results of the present study are akin to the published findings recorded 100% larval mortality after 48 h of incubation in 10% culture filtrate of the *Photorhabdus* spp. though the duration of incubation was longer than in the present study [30].

In this study, an attempt was made to assess the nematodicidal effect of isolates using a simulation pasture model which showed an 87.5% reduction in L_3_ for *Citrobacter amalonaticus*. The nematicidal effect of the bacterial isolates can be understood by comparing the reduction in L_3_ larvae observed in the current study with the findings of a previous study, in which 68 to 98 percent of third-stage Strongyle nematode larvae were recovered from grass samples collected from untreated, laboratory-maintained pastures [31].

The findings of the present study are in confirmation with reports of earlier workers. The researchers attributed the nematode-killing effect of bacteria to the release of a wide variety of products including antibiotics, toxins, lytic enzymes, secondary metabolites and volatile organic compounds [32]. In addition some bacteria directly attach to the nematode’s cuticles and proliferate within their body and kill them [33]. Additionally, *Chryseobacterium nematophagum* degrades the chitin lining of the foregut within 3 h of ingestion, enters the body cavity, and ultimately kills the nematode within 24 h, while *Bacillus thuringiensis* produces crystalline (Cry) proteins that damage the internal organs of various types of plant parasitic nematodes [19,34]. Hence, it could be presumed that the bacterial isolates of the present study might have possessed any one of the above traits or in combinations that caused nematode death within a short period of time. This approach primarily aims to reduce the number of infective L_3_ available to the grazing animals; thereby, GIN infection-associated economic losses can be minimized to a larger extent. Biological control can be categorized into direct and indirect approaches. Direct biological control refers to the use of organisms that act directly against nematodes, such as fungi, bacteria, and predatory or antagonistic nematodes. Indirect biological control refers to organisms, including earthworms and dung beetles, that contribute through their activities to the destruction of the parasites’ habitats [35].

As hitherto no study has been undertaken on assessing the nematodicidal effect of bacterial isolates under a pasture model, based on the results obtained in this trial, it could be presumed that the isolates of the present study can maintain their nematodicidal activity under field conditions. This observation may help in the formulation of a suitable bacterial product to use under field conditions to control pre-parasitic stages of strongyle nematodes. It is strongly believed that this approach may go a long way in controlling gastrointestinal nematode infections in small ruminants.

## 5. Conclusions

In this study, based on the results obtained, three bacterial isolates from animal sources exhibited a strong nematodicidal activity against strongyle nematodes of small ruminants. Among these, the isolate *Citrobacter amalonaticus* showed a higher level of larval mortality in a dose-dependent assay and simulation model study. However, using it as a broth culture under field conditions may have its own limitations such as administration and maintaining shelf life. Hence, it is concluded that a bacteria-based feed mix or freeze-dried powder formulation could be developed for convenient administration to small ruminants. Given the nematodicidal effect of these isolates, feeding small ruminants with a bacterial feed mix/powder will be an effective alternative to chemical anthelmintics for the prevention of parasitic gastroenteritis in small ruminants through controlling the pre-parasitic nematodes in the pasture.

## Figures and Tables

**Figure 2:**
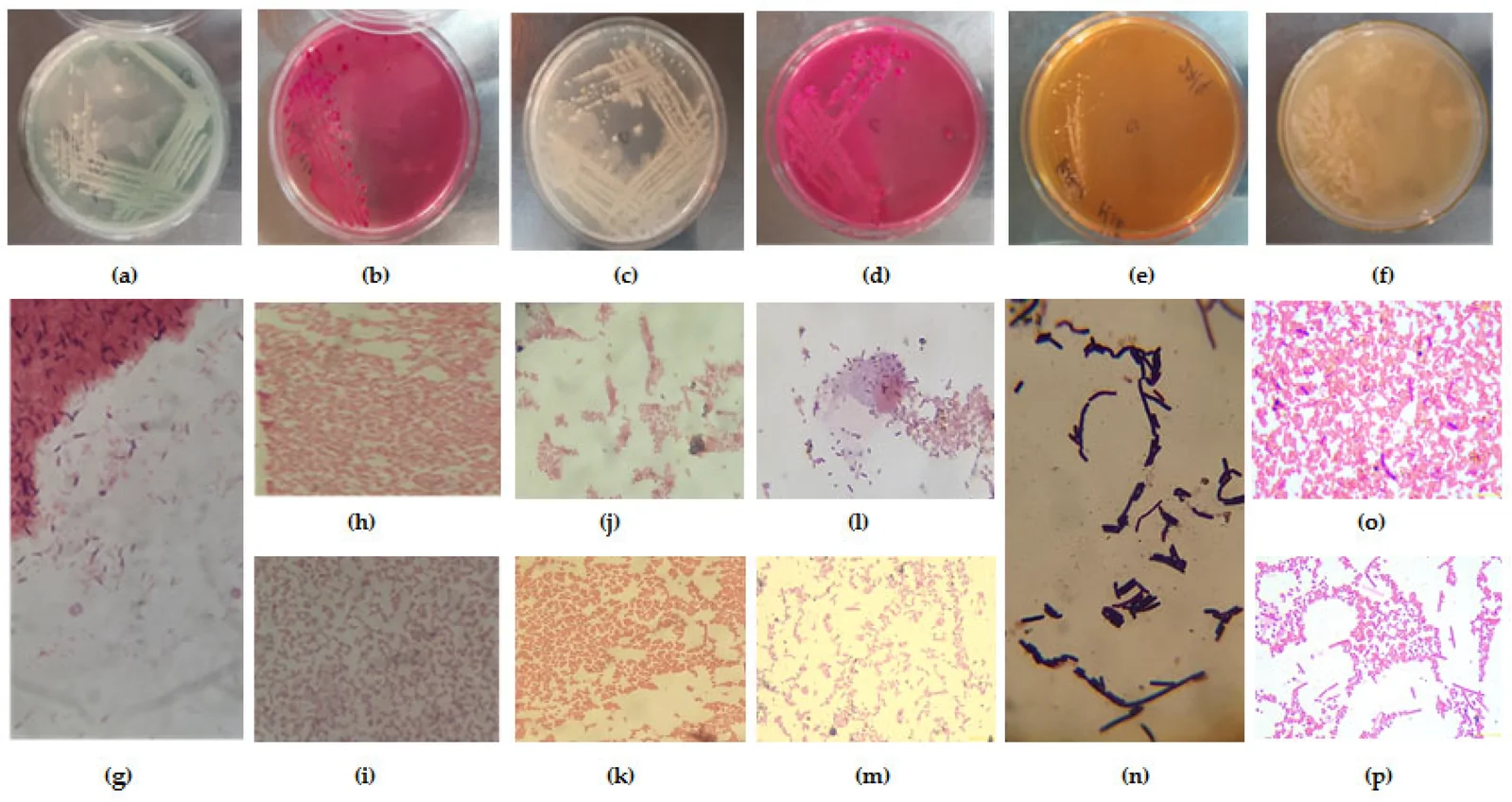
The morphological and cultural characterizations of bacterial isolates: (**a**) nutrient agar, white color colonies; (**b**) MacConkey agar, pale and pink color colonies; (**c**) nutrient agar, swarming growth, round and individual colonies; (**d**) MacConkey agar, pale and pink color colonies; (**e**) Mannitol salt agar, yellow color, dew drop colonies; (**f**) brain–heart infusion agar, swarming growth, round and individual colonies; (**g**–**i**) Gram-negative, rod; (**j**,**k**) Gram-positive, cocci; (**i**–**m**) Gram-negative, rod; (**n**) Gram-positive, rod; (**o**,**p**) Gram-negative, rod.

**Figure 3:**
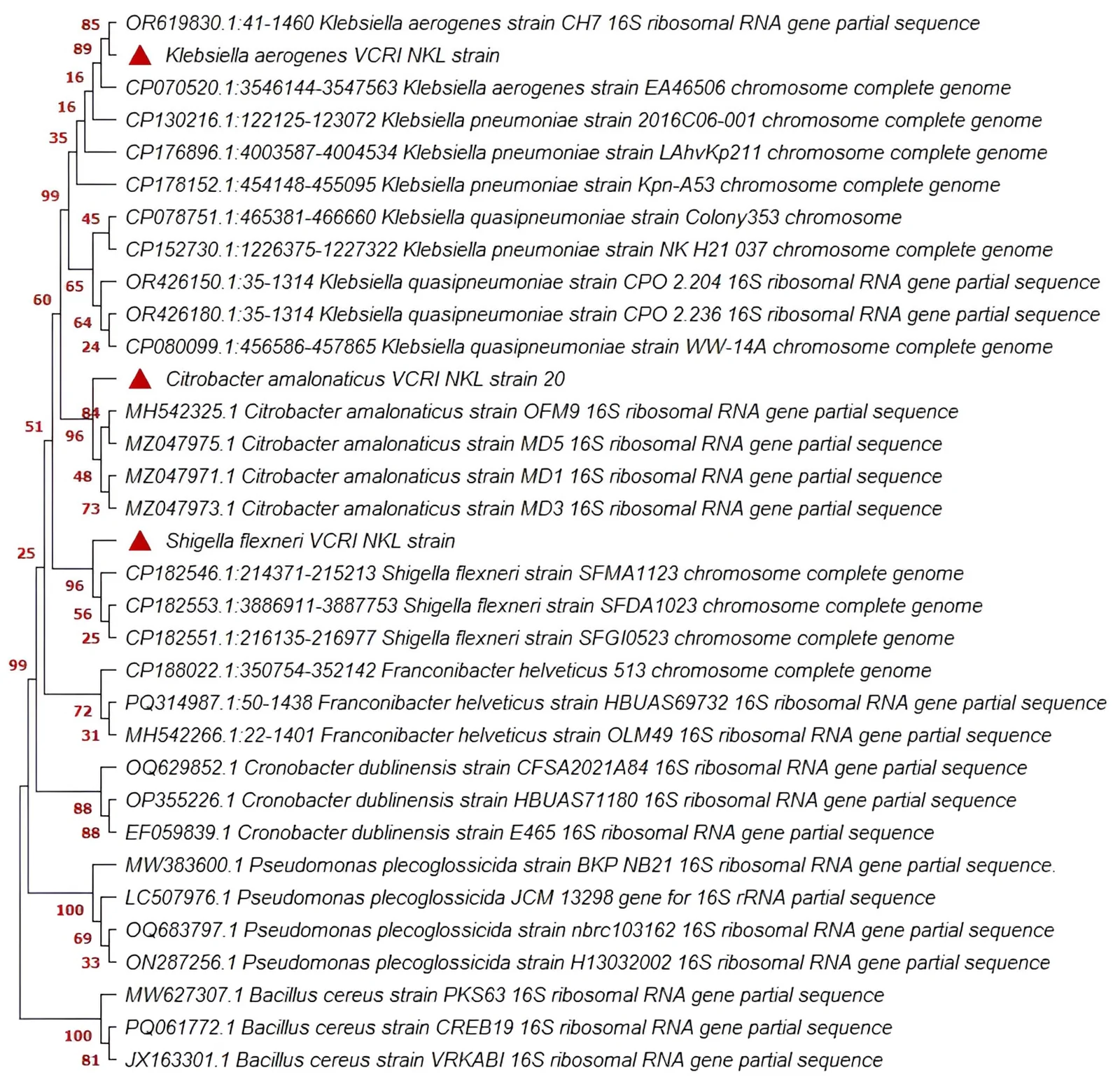
Phylogenetic tree for bacterial isolates ▲C2MCPK, ▲C2MCPL and ▲C2BHSY on their 16S rRNA gene sequences.

**Figure 4:**
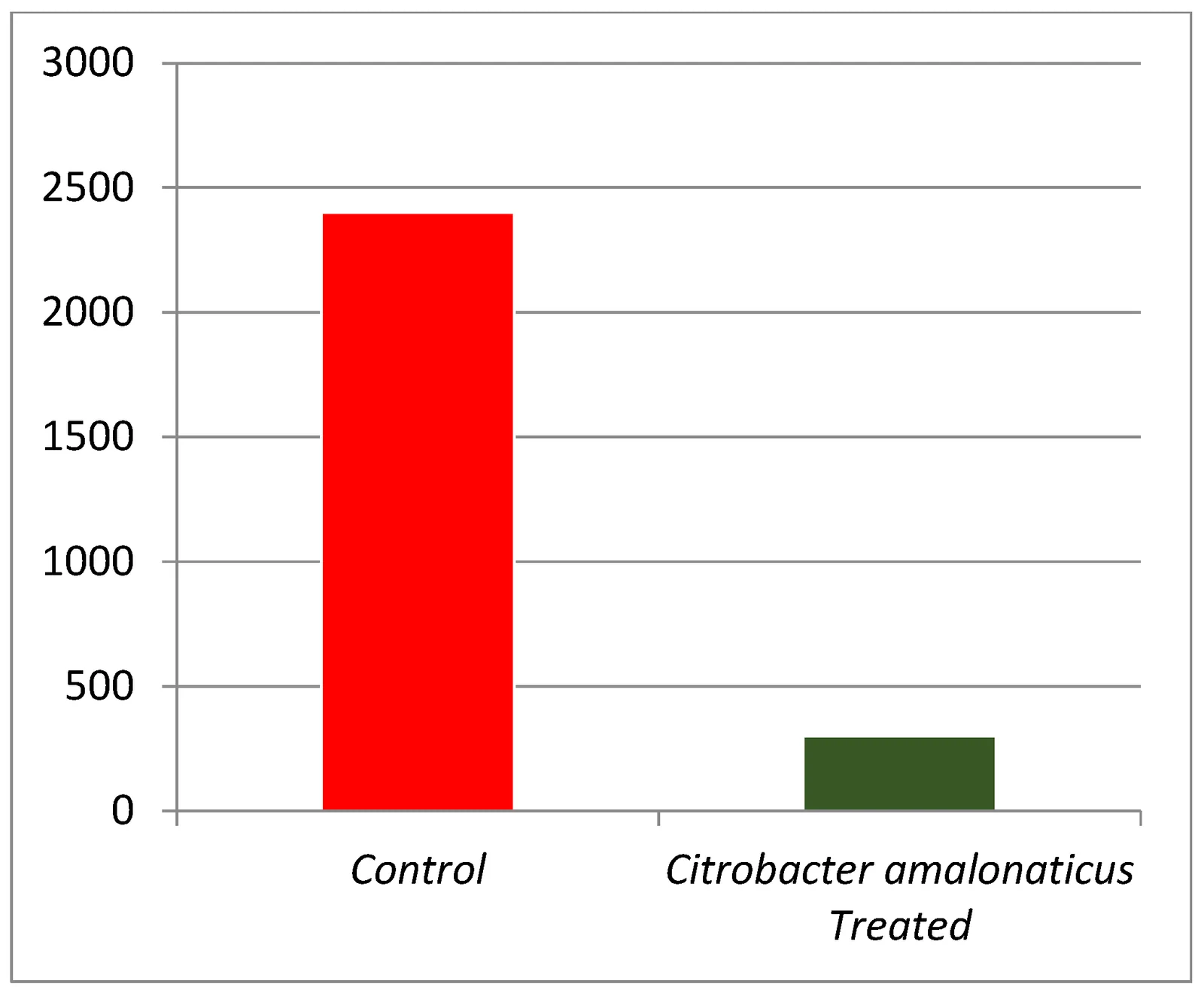
The number of L_3_/Kg of grass in simulation pasture model study.

**Table 1:** Colony morphology and Gram-staining characteristics of bacterial isolates.

Bacterial Isolates	Culture Characteristics of Isolates	Characteristics of Isolates in Gram Staining
C1MCPL ^1^	Pale color colonies	Gram-negative, Rod
C1MCPK ^1^	Pink color colonies	Gram-negative, Rod
C1NIC ^1^	White color colonies	Gram-negative, Rod
C2NTR ^1^	Swarming growth	Gram-positive, Cocci
C2NIC ^1^	Round, individual colonies	Gram-positive, Cocci
C2MS ^1^	Yellow color, dew drop colonies	Gram-positive, Rod
C2MCPK ^1^	Pink color colonies	Gram-negative, Rod
C2MCPL ^1^	Pale color colonies	Gram-negative, Rod
C2BHSY ^1^	Swarming growth	Gram-negative, Rod
C2BHIC ^1^	Round, individual colonies	Gram-negative, Rod

^1^ C1—cattle1; C2 – Cattle2; N—nutrient agar; MC—MacConkey agar; MS—Mannitol salt agar; BH—brain–heart infusion agar; I.C—individual colony; SY—swarming growth; PK—pink; PL—pale.

**Table 2:** Nematodicidal activity of larval feeding assay of bacterial isolates.

Bacterial Isolates	Percentage of Larval Mortality in Different Time Intervals				
	30 min	1 h	2 h	4 h	6 h
C1MCPL ^1^	9.33 ± 0.67 ^bc^	10.33 ± 0.88 ^bcd^	33.67 ± 2.19 ^c^	84.33 ± 0.88 ^b^	94.00 ± 1.00 ^c^
C1MCPK ^1^	0.00 ± 0.00 ^a^	20.33 ± 1.76 ^e^	91.00 ± 0.00 ^e^	97.00 ± 1.15 ^de^	100.00 ± 0.00 ^e^
C1NIC ^1^	26.33 ± 9.06 ^c^	68.67 ± 4.81 ^f^	89.67 ± 3.84 ^e^	89.67 ± 3.84 ^c^	97.33 ± 2.18 ^d^
C2NTR ^1^	10.67 ± 1.45 ^b^	24.33 ± 2.73 ^e^	65.00 ± 1.15 ^d^	99.00 ± 0.00 ^de^	99.00 ± 0.00 ^de^
C2NIC ^1^	0.00 ± 0.00 ^a^	5.67 ± 2.33 ^abc^	15.67 ± 4.91 ^b^	95.00 ± 2.65 ^d^	100.00 ± 0.00 ^e^
C2MS ^1^	0.00 ± 0.00 ^a^	0.00 ± 0.00 ^a^	0.00 ± 0.00 ^a^	0.00 ± 0.00 ^a^	0.00 ± 0.00 ^a^
C2MCPK ^1^	0.00 ± 0.00 ^a^	4.33 ± 1.45 ^ab^	98.67 ± 0.88 ^g^	100.00 ± 0.00 ^e^	100.00 ± 0.00 ^e^
C2MCPL ^1^	0.00 ± 0.00 ^a^	14.67 ± 3.93 ^de^	91.67 ± 3.18 ^d^	100.00 ± 0.00 ^e^	100.00 ± 0.00 ^e^
C2BHSY ^1^	0.00 ± 0.00 ^a^	12.33 ± 0.88 ^cd^	98.33 ± 0.67 ^fg^	100.00 ± 0.00 ^de^	100.00 ± 0.00 ^e^
C2BHIC ^1^	8.00 ± 1.15 ^ab^	26.00 ± 2.08 ^e^	98.33 ± 0.33 ^fg^	99.67 ± 0.33 ^e^	100.00 ± 0.00 ^e^
CONTROL (Water)	0.00 ± 0.00 ^a^	0.00 ± 0.00 ^a^	1.67 ± 0.88 ^a^	2.00 ± 1.00 ^a^	2.33 ± 0.67 ^b^
CONTROL (Probiotic)	0.00 ± 0.00 ^a^	0.00 ± 0.00 ^a^	0.00 ± 0.00 ^a^	0.00 ± 0.00 ^a^	0.00 ± 0.00 ^a^

Means ± S.E. bearing different superscripts differed significantly according to Duncan’s multiple range test at *p* < 0.05. ^1^ C1—cattle1; C2 – Cattle2; N—nutrient agar; MC—MacConkey agar; MS—Mannitol salt agar; BH—brain–heart infusion agar; PK—pink; PL—pale; TR—tree-like growth; I.C—individual colony; SY—swarming growth.

**Table 3:** Dose-dependent maximum larval mortality of *Citrobacter amalonaticus*.

Concentration of Culture Broth (%)	Percentage of Larval Mortality in Different Time Intervals					
	30 min	1 h	2 h	4 h	6 h	24 h
10	8.00 ± 0.58 ^c^	89.00 ± 1.15 ^d^	96.67 ± 0.33 ^d^	100.00 ± 0.00 ^e^	100.00 ± 0.00 ^c^	100.00 ± 0.00 ^c^
7.5	6.00 ± 2.31 ^bc^	65.00 ± 8.08 ^c^	92.00 ± 3.46 ^d^	98.67 ± 0.88 ^e^	100.00 ± 0.00 ^c^	100.00 ± 0.00 ^c^
5	2.00 ± 1.15 ^ab^	73.33 ± 3.18 ^cd^	71.67 ± 4.63 ^c^	93.33 ± 0.33 ^d^	97.67 ± 2.33 ^c^	100.00 ± 0.00 ^c^
2.5	6.33 ± 2.33 ^bc^	21.00 ± 9.24 ^b^	24.33 ± 6.74 ^b^	40.00 ± 0.58 ^c^	21.67 ± 9.74 ^b^	26.00 ± 10.82 ^b^
1.25	0.00 ± 0.00 ^a^	5.00 ± 1.73 ^ab^	8.33 ± 0.67 ^a^	14.00 ± 2.31 ^b^	15.67 ± 2.03 ^b^	21.33 ± 4.91 ^b^
CONTROL (Water)	0.00 ± 0.00 ^a^	0.00 ± 0.00 ^a^	0.00 ± 0.00 ^a^	0.00 ± 0.00 ^a^	8.67 ± 0.88 ^b^	12.33 ± 2.60 ^b^
CONTROL (Probiotic)	0.00 ± 0.00 ^a^	0.00 ± 0.00 ^a^	0.00 ± 0.00 ^a^	0.00 ± 0.00 ^a^	0.00 ± 0.00 ^a^	0.00 ± 0.00 ^a^

Means ± S.E. bearing different superscripts differed significantly according to Duncan’s multiple range test at *p* < 0.05.

## Data Availability

The original contributions presented in this study are included in the article. Further inquiries can be directed to the corresponding authors.
